# Anti-inflammatory effects of dexamethasone and meloxicam on *Borrelia burgdorferi*-induced inflammation in neuronal cultures of dorsal root ganglia and myelinating cells of the peripheral nervous system

**DOI:** 10.1186/s12974-015-0461-y

**Published:** 2015-12-23

**Authors:** Geeta Ramesh, Olivia C. Meisner, Mario T. Philipp

**Affiliations:** Division of Bacteriology and Parasitology, Tulane National Primate Research Center, Covington, LA USA; Department of Neuroscience and Behavioral Biology, Emory College of Arts and Sciences, Atlanta, GA USA; Department of Microbiology and Immunology, Tulane University Medical School, New Orleans, LA USA

**Keywords:** Lyme neuroborreliosis, Inflammation, Apoptosis, Dorsal root ganglia, Schwann cells, Dexamethasone, Meloxicam

## Abstract

**Background:**

Lyme neuroborreliosis (LNB), caused by the spirochete *Borrelia burgdorferi* (Bb), could result in cognitive impairment, motor dysfunction, and radiculoneuritis. We hypothesized that inflammation is a key factor in LNB pathogenesis and recently evaluated the effects of dexamethasone, a steroidal anti-inflammatory drug, and meloxicam a non-steroidal anti-inflammatory drug (NSAID), in a rhesus monkey model of acute LNB. Dexamethasone treatment significantly reduced the levels of immune mediators, and prevented inflammatory and/or neurodegenerative lesions in the central and peripheral nervous systems, and apoptosis in the dorsal root ganglia (DRG). However, infected animals treated with meloxicam showed levels of inflammatory mediators, inflammatory lesions, and DRG cell apoptosis that were similar to that of the infected animals that were left untreated.

**Methods:**

To address the differential anti-inflammatory effects of dexamethasone and meloxicam on neuronal and myelinating cells of the peripheral nervous system (PNS), we evaluated the potential of these drugs to alter the levels of Bb-induced inflammatory mediators in rhesus DRG cell cultures and primary human Schwann cells (HSC), using multiplex enzyme-linked immunosorbent assays (ELISA). We also ascertained the ability of these drugs to modulate cell death as induced by live Bb in HSC using the 3-(4,5-dimethylthiazol-2-yl)-2,5-diphenyltetrazolium bromide (MTT) viability assay and the potential of dexamethasone to modulate Bb-induced apoptosis in HSC by the TUNEL assay.

**Results:**

Earlier, we reported that dexamethasone significantly reduced Bb-induced immune mediators and apoptosis in rhesus DRG cell cultures. Here, we report that dexamethasone but not meloxicam significantly reduces the levels of several cytokines and chemokines as induced by live Bb, in HSC and DRG cell cultures. Further, meloxicam does not significantly alter Bb-induced cell death in HSC, while dexamethasone protects HSC against Bb-induced cell death.

**Conclusions:**

These data help further explain our in vivo findings of significantly reduced levels of inflammatory mediators, DRG-apoptosis, and lack of inflammatory neurodegenerative lesions in the nerve roots and DRG of Bb-infected animals that were treated with dexamethasone, but not meloxicam. Evaluating the role of the signaling mechanisms that contribute to the anti-inflammatory potential of dexamethasone in the context of LNB could serve to identify therapeutic targets for limiting radiculitis and axonal degeneration in peripheral LNB.

## Background

Lyme disease is caused by infection with the spirochete *Borrelia burgdorferi* (Bb) [1]. Nervous system involvement in Lyme disease, termed Lyme neuroborreliosis (LNB), is manifested in about 15 % of Lyme disease patients and may affect both the central and peripheral nervous systems. Patients with LNB typically show the neurological triad of meningitis, cranial neuritis, and radiculoneuritis, commonly described as meningoradiculitis (a.k.a. Garin-Bujadoux-Bannwarth’s syndrome) [1–8].

Radiculitis or radiculoneuritis that presents as neurogenic pain along the back, radiating into the legs and foot, with numbness and tingling in the legs, is the most common manifestation in patients with peripheral LNB [9–11]. Polyneuritis affecting multiple cranial nerves may occur, presenting as facial palsy, optic neuritis and uveitis, abnormalities in ocular, acoustic, and taste reflexes, and aphasia [12–16]. Pathology examinations in patients with peripheral nervous system (PNS) Lyme disease have shown inflammation in the nerve roots and dorsal root ganglia (DRG) and patchy multifocal axonal loss accompanied with epineural perivascular inflammatory infiltrates or perineuritis [10, 17, 18]. Patients exhibiting electrophysiological abnormalities indicative of widespread axonal damage, and nerve conduction slowing, with abnormal temporal dispersion consistent with demyelinating neuropathy, have also been reported in LNB [10, 11, 19–22].

The rhesus macaque is an accurate model of human nervous system Lyme disease [23–27]. Infection in nerve roots, DRG, and sensory ganglia showing varying degrees of necrosis, with peripheral nerve specimens showing multifocal axonal degeneration and regeneration, and nerve conduction study results consistent with mononeuropathy multiplex have all been observed in the rhesus monkey model of LNB [28]. Previously, we reported that acute neurological manifestations identified histopathologically as leptomeningitis and radiculitis were concomitant with the inflammatory response elicited by the Lyme disease spirochete [27].

We hypothesized that inflammation induced by Bb is a key factor in mediating LNB pathogenesis. We recently evaluated the inflammatory changes in rhesus macaques infected with Bb that were either left untreated or were given the anti-inflammatory drug dexamethasone, a steroid that inhibits the expression of several immune mediators [29] or meloxicam, the non-steroidal anti-inflammatory drug (NSAID) that inhibits cyclooxygenase-2 (COX-2) [30]. Importantly, dexamethasone treatment significantly reduced the levels of immune mediators (IL-6, IL-8, CCL2, and CXCL13) and pleocytosis in the cerebrospinal fluid (CSF), and prevented inflammatory and/or neurodegenerative lesions in the central and peripheral nervous systems, and apoptosis in the DRG [31]. Infected animals that were treated with meloxicam showed only a significant reduction in the levels of the B cell chemokine CXCL-13 in the CSF, but the levels of other immune mediators and the inflammatory lesions displayed were similar to that of infected animals that were left untreated.

Our observations suggested that dexamethasone and meloxicam had differential anti-inflammatory effects on Bb-induced inflammation in glial and neuronal cells. Earlier, we reported that dexamethasone significantly reduced Bb-induced immune mediators and apoptosis in rhesus DRG cell cultures [32]. To address this notion further, we evaluated the ability of dexamethasone and meloxicam to modulate the levels of inflammatory mediators and cell death as induced by live Bb in neuronal cells and myelinating cells of the PNS. We specifically evaluated the potential of these drugs to alter the levels of Bb-induced inflammatory mediators in culture supernatants of primary rhesus DRG cell cultures and human Schwann cells (HSC), using multiplex enzyme-linked immunosorbent assays (ELISA). We also ascertained the ability of these drugs to modulate cell death as induced by live Bb in HSC using the 3-(4,5-dimethylthiazol-2-yl)-2,5-diphenyltetrazolium bromide (MTT) viability assay and the potential of dexamethasone to modulate Bb-induced apoptosis in HSC by the in situ terminal deoxynucleotidyl transferase dUTP nick end labeling (TUNEL) assay. Here, we report that dexamethasone but not meloxicam significantly reduces the levels of several cytokines and chemokines as induced by live Bb in HSC and DRG cell cultures. Further, meloxicam does not significantly alter Bb-induced cell death in HSC, while dexamethasone protects HSC against Bb-induced cell death.

## Methods

### Growth and preparation of live spirochetes

*B. burgdorferi* B31 clone 5A19 spirochetes, passages 1–3 were grown in Barbour-Stoenner-Kelly (BSK) medium, supplemented with 6 % rabbit serum (Sigma, St. Louis, MO) and antibiotics (rifampicin at 45.4 μg/mL, phosphomycin at 193 μg/mL, and amphotericin at 0.25 μg/mL) to late logarithmic phase under microaerophilic conditions. Spirochetes were pelleted, washed using sterile phosphate-buffered saline (PBS), and resuspended in the working medium at the desired density, as previously described [32].

### Rhesus primary DRG cultures

Primary rhesus DRG cell cultures were derived from adult rhesus DRG tissues obtained fresh at necrospy, as described earlier [32].

### HSC cultures

Cryopreserved HSC obtained from ScienCell Inc. (Carlsbad, CA) were cultured as previously described [32].

### Immunofluorescence staining and confocal microscopy for the expression of MBP in Schwann cell cultures

Medium was removed from HSC cultures and cells were fixed in 2 % paraformaldehyde (PFA) followed by post-fixation permeabilization using a mixture of ethanol:acetic acid (2:1) (Sigma) for 5 min at −20 °C and detached from the chamber and processed for immunofluorescence staining as previously described [32]. Schwann cell cultures were stained for the expression of myelin basic protein (MBP), using rabbit polyclonal anti-human MBP Clone AB 980 at 1:100 (Millipore, Billerica, MA) and the relevant secondary antibody, goat anti-rabbit conjugated to one of the Alexa fluorochromes, Alexa-568 (Invitrogen) at a dilution of 1:1000 as previously described [32]. The stained and mounted slides were stored in the dark at 4 °C until they were viewed. Confocal microscopy was performed using a Leica TCS SP2 confocal microscope equipped with three lasers (Leica Microsystems, Exton, PA). Images of individual channels were merged to obtain images containing all channels.

### Evaluating the anti-inflammatory potential of dexamethasone and meloxicam in DRG and HSC cultures stimulated with live Bb

Bb strain B31 5A19 passage 3 was prepared as described above. The DRG and HSC cultures were washed in their respective media, devoid of antibiotics. The Bb cells were resuspended in culture medium devoid of antibiotics, at the desired multiplicity of infection (MOI). Controls with no spirochetes were also included. Prior to stimulation with live Bb, DRG cell cultures and HSC cultures were incubated with various concentrations of dexamethasone (water soluble), 5, 15, and 150 μM (Sigma), or meloxicam (water soluble), 1, 10, 50, and 100 μM (Sigma) for 2 h at 37 °C, after which they were washed and then incubated in fresh growth medium containing the respective concentrations of dexamethasone or meloxicam and live Bb at a MOI of 10:1 at 37 °C. Similar concentrations of dexamethasone as those mentioned above have been reported to inhibit the production of CCL2 in mice microglia [33]. Meloxicam at a concentration range of 1–100 μM has been previously shown to be effective in inhibiting COX-2 in in vitro studies [34].

The DRG cell cultures were incubated for 24 h as described earlier [32], while HSC cultures were incubated for 48 h, post-infection, respectively, in a humidified 5 % CO_2_ incubator, set at 37 °C. At the end of the incubation times, culture supernatants were collected for evaluation of inflammatory mediators. Culture supernatants were centrifuged at 4 °C at 2000×*g* to remove any suspended bacteria, and the supernatant was aliquoted and stored at −70 °C until used.

### Evaluation of immune mediators from culture supernatants

The concentrations of cytokines and chemokines present in the culture supernatants from rhesus DRG were quantified using the MilliPlex MAP Non-Human Primate Cytokine Magnetic Bead Panel—Premixed 23 Plex, Cytokine-Chemokine Array kit (Millipore), following the manufacturer’s instructions. The analytes detected by this panel are G-CSF, GM-CSF, IFN-γ, IL-10, IL-12/23 (p40), IL-13, IL-15, IL-17, IL-18, IL-1ra, IL-1β, IL-2, IL-4, IL-5, IL-6, IL-8, CCL2, CCL3, CCL4, TGF-α, TNF, VEGF, and sCD40L. The concentrations of cytokines and chemokines present in the culture supernatants from HSC cultures described above were quantified using the Bio-Plex Pro™ Human Cytokine 27-plex Assay (BioRad, Hercules, CA). The analytes detected by this panel are FGF basic, Eotaxin, G-CSF, GM-CSF, IFN-γ, IL-1β, IL-1ra, IL-2, IL-4, IL-5, IL-6, IL-7, IL-8, IL-9, IL-10, IL-12 (p70), IL-13, IL-15, IL-17, IP-10, MCP-1 (CCL-2), MIP-1alpha, MIP-1beta, PDGF-BB, RANTES, TNF, and VEGF. The multiplex plate was read using a Bio-Plex 200 Suspension Array Luminex System (Bio-Rad).

### Evaluation of the ability of live Bb spirochetes to induce HSC death by the MTT cell viability assay

HSC cell cultures were seeded as described earlier [32] on poly-L-Lysine-coated 6-well plates and maintained in growth medium for 48 h. Cultures were incubated with live Bb at MOI of 5:1, 10:1, and 50:1 or left in medium alone for a period of 48 h, after which viability of HSC was evaluated by the MTT cell viability assay using the tetrazolium dye MTT as a substrate, and conducted according to manufacturer’s protocols (Sigma). Briefly, 5 mg/mL of MTT reagent was added at a volume corresponding to 10 % of the volume of medium in the wells and incubated at 37 °C, 5 % CO2 for 2 h. Cells were solubilized using the “solubilization solution” and the optical density of the resulting colored solution was read at 570 nm, spectrophotometrically.

### Evaluation of HSC apoptosis by the in situ TUNEL assay

Cells contained in chamber slides were labeled for MBP by immunofluorescence staining as described above. Slides were then fixed with 2 % PFA, washed three times with PBS by rinsing slides with PBS and holding them in PBS for 2 min between washes. Slides were then subjected to the TUNEL-ApopTagPlus fluorescein in situ apoptosis assay (Chemicon, Temecula, CA) as per the manufacturer’s instructions. Slides were then mounted as described above and stored at 4 °C in the dark until viewed. The percentage of apoptotic cells from ten fields was evaluated from each chamber area by counting the total number of MBP-positive cells (at least 500 cells) from each of the chamber areas, followed by the percentage of cells that showed co-localization of both the TUNEL signal and MBP expression. All counts were made by viewing slides under a fixed magnification of 63 x (corresponding to an area of 0.05 mm^2^) using the confocal microscope.

### Evaluation of the protective effect of dexamethasone on Bb-induced apoptosis in HSC cultures

HSC cell cultures were seeded as described above in chamber slides for evaluation of apoptosis or for evaluation of immune mediators and maintained in growth medium for 48 h. Prior to stimulation with live Bb, HSC cultures were incubated with various concentrations of dexamethasone (5, 15, and 150 μM) for 2 h at 37 °C, after which they were washed and then incubated in fresh growth medium containing the respective concentrations of dexamethasone and live Bb at a MOI of 10:1 at 37 °C for 48 h and devoid of antibiotics.

After 48 h, culture cells were fixed, stained for MBP by immunofluorescence staining, and evaluated for apoptosis by the in situ TUNEL assay as described above. Medium controls that were pretreated and then incubated with the same respective concentrations of dexamethasone but without the addition of live Bb were also included.

### Statistical evaluation

The one-way ANOVA and Tukey’s multiple comparison test was used to evaluate the statistical significance between means of data sets, using Graphpad Prizm software (Graph Pad Software Inc.) version 5.

## Results

### Dexamethasone but not meloxicam significantly reduces the levels of cytokines and chemokines induced by live Bb in rhesus DRG cell cultures

**Fig. 1:**
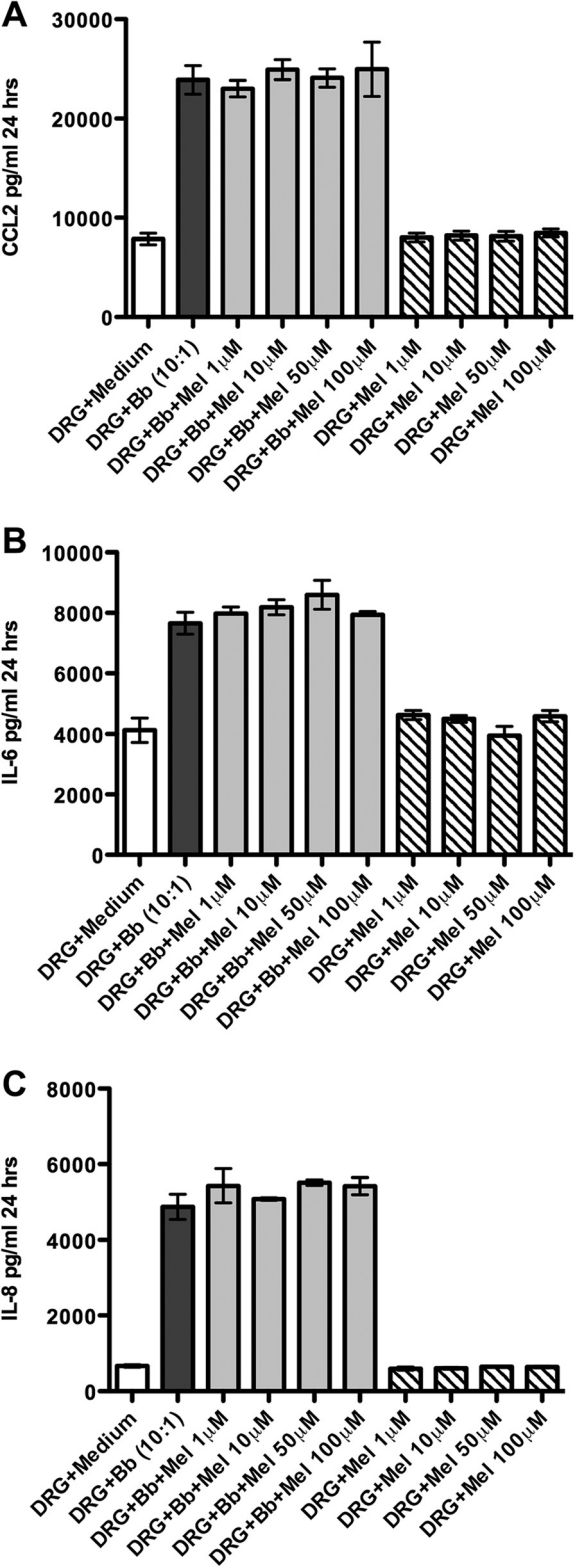
Meloxicam does not significantly alter levels of Bb-induced cytokines and chemokine in DRG cell cultures. The effect of meloxicam (Mel) on the levels of CCL2 (**a**), IL-6 (**b**), and IL-8 (**c**) as induced by live Bb in rhesus DRG cell cultures. Data represent mean and SEM of values from DRG cell cultures established from three adult rhesus macaques. The one-way ANOVA and Tukey’s multiple comparison test was used to evaluate the statistical significance between means of data sets

We have reported that the anti-inflammatory drug dexamethasone is able to significantly reduce the levels of CCL2, IL-6, and IL-8 as induced by live Bb at a MOI of 10:1 in DRG cell cultures, after 24 h in a dose-dependent fashion [32]. Here, we report, in contrast, that meloxicam did not show any significant alteration in the levels of CCL2, IL-6, and IL-8 in DRG cell cultures that were exposed to live Bb at the concentrations tested, Fig. 1a–c, respectively. Data represent mean and SEM of values from DRG cell cultures established from three adult rhesus macaques.

### Dexamethasone but not meloxicam significantly reduces the levels of cytokines and chemokines induced by live Bb in HSC

Earlier, we had reported on the ability of live Bb to induce CCL2, IL-6, and IL-8 in HSC [32]. Here, we report that culture supernatants of HSC that were incubated with live Bb at a MOI of 10:1 for 48 h show significantly elevated levels of several cytokines and chemokines as compared to medium controls, in addition to CCL2, IL-6, and IL-8 when evaluated by the Pro™ Human Cytokine 27-plex Assay (BioRad), as shown in Table 1. IL-8, VEGF, CCL-2, IL-6, and G-CSF reached the highest levels, while IP-10, IL-12, IFN-γ, IL-1RA, IL-10, IL-15, CCL-5, PDGF, and TNF reached relatively lower levels though significantly elevated as compared to medium controls.

**Table 1:** Effect of dexamethasone and meloxicam on cytokines and chemokines induced by live Bb in HSC

Immune mediator	HSC medium control	HSC + live Bb (10:1)	HSC + live Bb + dexamethasone (150 μM)	HSC + live Bb + meloxicam (100 μM)
IL-1RA	31.38 ± 0.97	84.76 ± 9.75	18.10 ± 0.97	130.09 ± 29.18
			*p* < 0.001	*p* < 0.05
IL-6	266.62 ± 12.75	1420.76 ± 62.98	850.96 ± 19.09	1845.76 ± 23.87
			*p* < 0.001	*p* < 0.001
IL-8	1930.90 ± 129.43	10297.62 ± 759.93	3093.81 ± 198.68	13,686.96 ± 494.18
			*p* < 0.001	*p* < 0.001
IL-10	34.86 ± 1.45	89.11 ± 2.23	110.68 ± 2.08	92.04 ± 11.43
			*p* < 0.001	ns
IL-12 p70	303.45 ± 21.69	658.21 ± 29.69	367.02 ± 15.02	767.67 ± 36.3
			*p* < 0.001	*p* < 0.05
IL-15	6.56 ± 0.58	23.67 ± 2.54	11.42 ± 1.34	38.49 ± 3.82
			*p* < 0.001	*p* < 0.001
CCL2/MCP-1	885.83 ± 51.57	3227.74 ± 153.35	835.10 ± 25.57	4719.75 ± 212.96
			*p* < 0.001	*p* < 0.001
CCL-5/RANTES	3.65 ± 0.7	38.19 ± 3.79	8.49 ± 1.79	47.99 ± 3.76
			*p* < 0.001	*p* < 0.01
PDGF	5.65 ± 1.04	37.84 ± 4.07	14.69 ± 1.74	40.38 ± 3.47
			*p* < 0.001	ns
IFN-γ	34.95 ± 5.48	86.33 ± 9.31	38.59 ± 3.54	112.06 ± 15.18
			*p* < 0.001	ns
IP-10	18.94 ± 2.75	856.47 ± 73.95	365.94 ± 24.53	853.49 ± 52.38
			*p* < 0.001	ns
G-CSF	511.26 ± 56.98	3032.42 ± 116.01	1725.49 ± 65.32	3410.18 ± 97.08
			*p* < 0.001	*p* < 0.05
VEGF	1088.41 ± 162.82	5925.06 ± 195.08	2920.06 ± 130.67	6943.79 ± 172.48
			*p* < 0.001	*p* < 0.001
TNF	5.24 ± 0.9	22.22 ± 2.79	8.15 ± 1.87	24.02 ± 2.69
			*p* < 0.001	ns

Cytokines and chemokines in HSC culture supernatants after 48 h of incubation with live Bb (MOI 10:1). Data represent mean (pg/ml) and SD of triplicate evaluations (*p* values represent significance for comparisons of values for Bb and Bb + Dex 150 μM or Bb and Bb + Mel 100 μM and *ns* not significant)

**Fig. 2:**
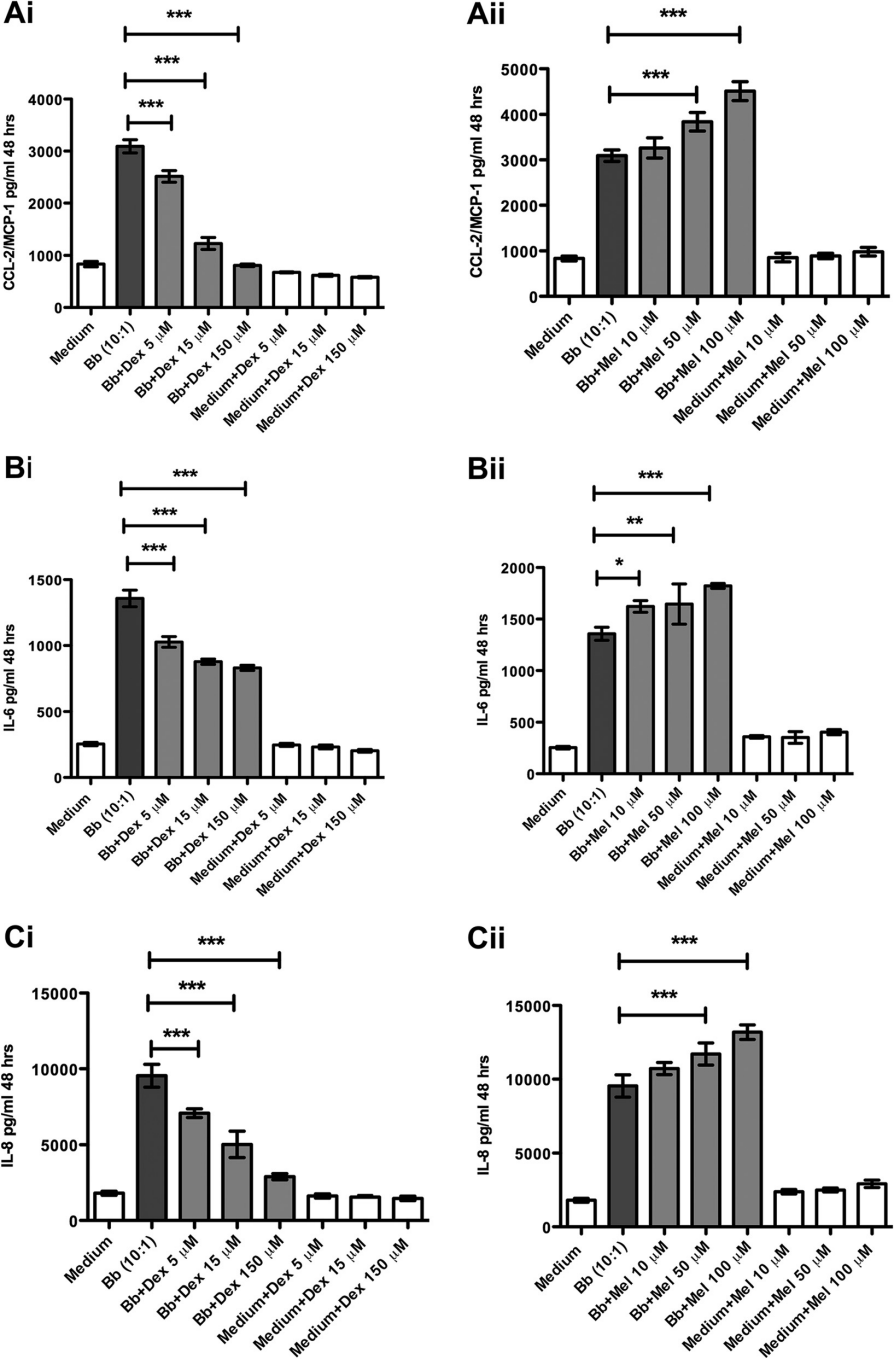
Dexamethasone but not meloxicam significantly reduces levels of Bb-induced cytokines and chemokines in HSC. The anti-inflammatory effect of dexamethasone (*Dex*) (**Ai**) and meloxicam (*Mel*) (**Aii**) on the levels of CCL2 as induced by live Bb in HSC. The anti-inflammatory effect of dexamethasone (**Bi**) and meloxicam (**Bii**) on the levels of IL-6 as induced by live Bb in HSC. The anti-inflammatory effect of dexamethasone (**Cii**) and meloxicam (**Ciii**) on the levels of IL-8 as induced by live Bb in HSC. The one-way ANOVA and Tukey’s multiple comparison test was used to evaluate the statistical significance between means and SD of triplicate data sets, *p < 0.05, **p < 0.01, ***p < 0.001

Dexamethasone but not meloxicam was able to significantly reduce the levels of the cytokines and chemokines in HSC, at the concentrations tested. This is shown in Table 1. Dexamethasone (150 μM) treatment resulted in significantly reduced levels of all of the immune mediators induced by live Bb, except IL-10, which showed a significantly elevated level as compared to that induced by Bb alone. Meloxicam treatment (100 μM) resulted in similar or slightly elevated levels of immune mediators elicited in response to Bb. A graphical representation of the dose-dependent effects of dexamethasone and meloxicam on the levels of the CCL2, IL-6, and IL-8 is shown in Fig. 2a–c, respectively. Data represent mean and SD of values from triplicate evaluations. While increasing concentrations of dexamethasone resulted in the expected gradual decrease in the levels of mediators, the effect of meloxicam was opposite, resulting in significantly elevated levels as the concentration of this drug was increased (Fig. 2 Ai–Cii).

### Dexamethasone but not meloxicam protects HSC from Bb-induced cell death

**Fig. 3:**
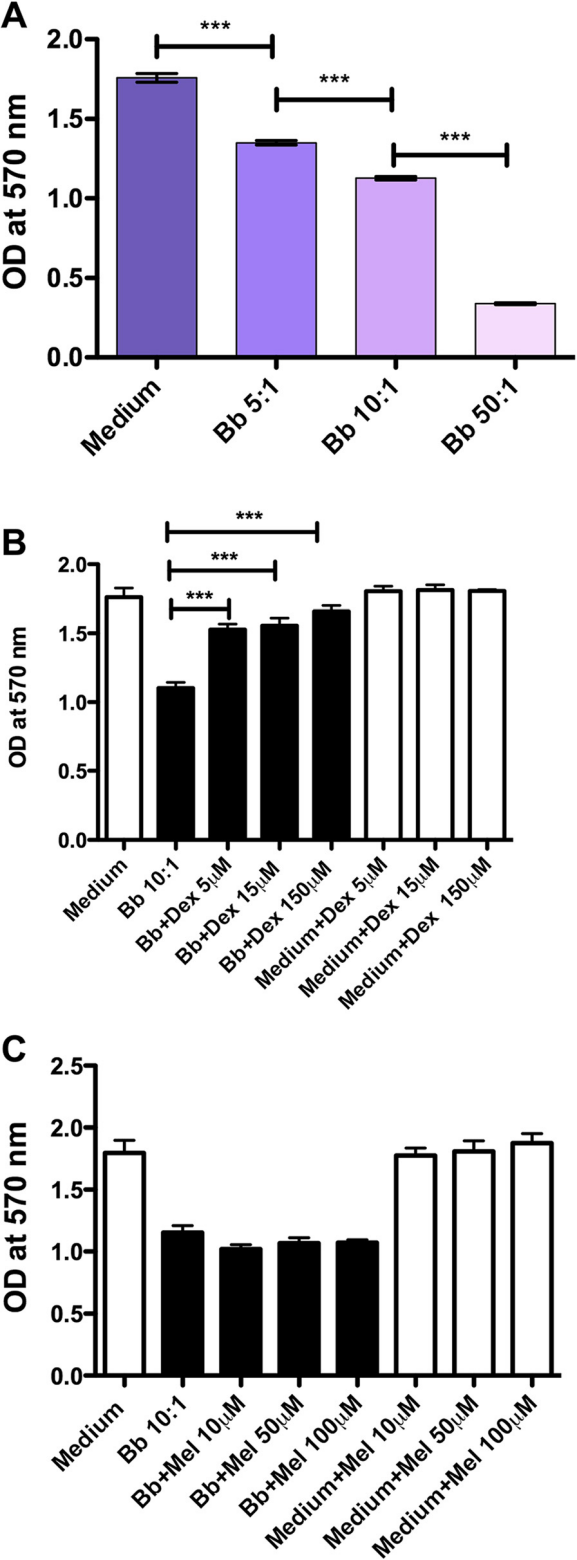
Dexamethasone but not meloxicam protects HSC from Bb-induced cell death. Cell viability as assayed by the MTT viability assay of HSC incubated with live Bb at various multiplicities of infection for 48 h is shown in **a**. The effect of dexamethasone on Bb-induced cell death as visualized by the MTT assay in HSC (**b**). The effect of meloxicam on Bb-induced cell death as visualized by the MTT assay in HSC (**c**). The one-way ANOVA and Tukey’s multiple comparison test was used to evaluate the statistical significance between means and SD of triplicate data sets, ***p < 0.001

Live Bb induces cell death in HSC in a dose-dependent fashion. Cell viability, as assayed by the MTT assay, of HSC incubated with Bb at various multiplicities of infection for 48 h is shown in Fig. 3a. The optical density of the HSC cultures as measured after incubation with the MTT substrate corresponds to the amount of purple formazan formed due to cleavage of MTT by mitochondrial dehydrogenase present only in viable cells. As the HSC are incubated with increasing MOI of live Bb, the optical density is significantly reduced compared to medium controls, signifying the increase in the proportion of dead cells in the culture.

Incubation of HSC with live Bb at a MOI of 10:1 in the presence of increasing concentrations of dexamethasone resulted in significant reduction in the amount of cell death as visualized by the MTT assay (Fig. 3b). However, meloxicam did not significantly alter the levels of cell death as induced by live Bb (Fig. 3c).

### Dexamethasone protects HSC from Bb-induced apoptosis in a dose-dependent fashion

**Fig. 4:**
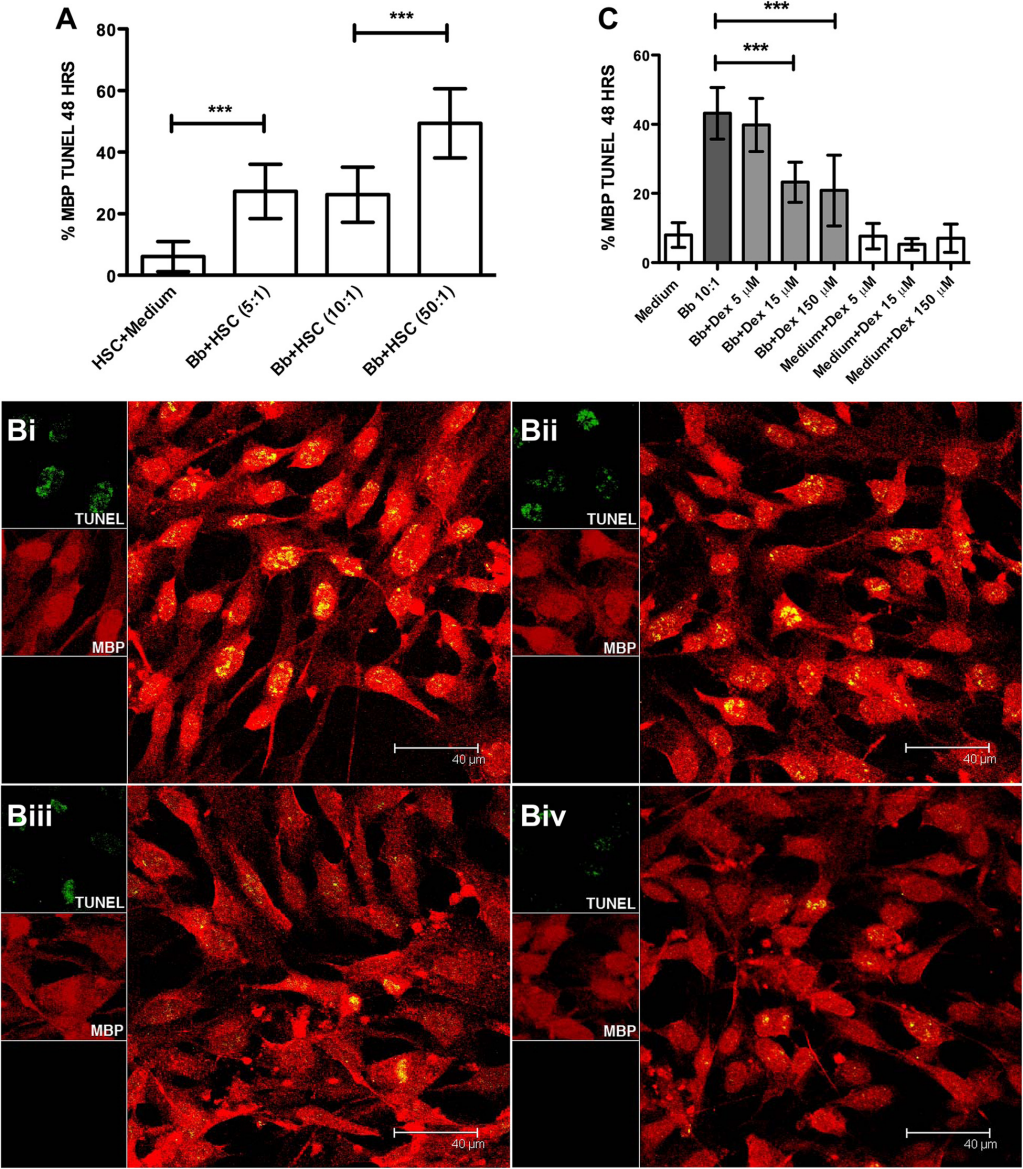
Dexamethasone protects HSC from Bb-induced apoptosis in a dose-dependent fashion. **A** Graphical representation of the percent apoptosis of HSC as induced by increasing MOI of live Bb. Representative images of the quantitative view of apoptosis after immunofluorescence staining and visualized by confocal microscopy by the in situ TUNEL assay (green) as induced by live Bb (MOI 10:1) in HSC stained with MBP (*red*), after 48 h, (**Bi**), Bb in the presence of 5 μM dexamethasone (**Bii**), 15 μM dexamethasone (**Biii**), and 150 μM dexamethasone (**Biv**), respectively. **C** Graphical representation of the effect of dexamethasone on the levels of apoptosis in HSC as induced by live Bb as visualized by the in situ TUNEL assay. The one-way ANOVA and Tukey’s multiple comparison test was used to evaluate the statistical significance between means and SD of ten data sets (approximately 500 cells) for each condition, ***p < 0.001

Apoptosis as measured by the TUNEL assay in HSC, with increasing MOIs of live Bb in culture is shown in Fig. 4A. Live Bb induced enhanced levels of apoptosis at a MOI of 5:1, as compared to that in medium controls after 48 h in culture. Levels of apoptosis increased further at a Bb MOI of 50:1. As we had observed that dexamethasone significantly reduced the levels of Bb-induced cytokines and chemokines in HSC, we ascertained if inflammation had a causal role in mediating apoptosis in HSC. We thus evaluated the effect of the anti-inflammatory drug dexamethasone on Bb-induced apoptosis. A representative confocal image of HSC apoptosis in the presence of live Bb at a MOI of 10:1 after 48 h of incubation, as visualized by the in situ TUNEL assay, is shown in Fig. 4Bi. The levels of protection from Bb-induced apoptosis increased with increasing concentrations of dexamethasone, 5 μM, Fig. 4Bii, 15 μM, Fig. 4Biii, and 150 μM, Fig. 4Biv. Figure 4C shows a graphical representation of the quantification of the levels of dexamethasone-protection described in Fig. 4B. Incubation of HSC with live Bb in the presence of dexamethasone (15 and 150 μM) resulted in significant reduction in the levels of apoptosis (Fig. 4C).

## Discussion

The pathogenesis of Lyme disease neuropathies is not well understood. The ability of Bb to induce cytokines, chemokines, and other inflammatory mediators in glial and neuronal cells, as well as glial and neuronal apoptosis has been well documented [27, 31, 32, 35–39]. Our recent observations in an in vivo model of acute LNB in the rhesus monkey suggest that dexamethasone and meloxicam have differential anti-inflammatory effects on Bb-induced inflammation in glial and neuronal cells [31]. We had previously reported that dexamethasone significantly reduced Bb-induced immune mediators and apoptosis in rhesus DRG cell cultures [32]. In this study, we explored the anti-inflammatory potential of meloxicam in these cells and the effects of both dexamethasone and meloxicam on primary HSC cultures in the presence and absence of Bb.

Radiculitis or inflammation of the dorsal root with neurogenic pain and altered sensory reflexes is commonly reported in adult patients with LNB [11]. The immune mediators IL-6, IL-8, and CCL2, which we found to be elevated in the DRG cell cultures exposed to live Bb, play a role in modulating inflammation in addition to the pain response [11, 40–43]. As the sensory neurons of the DRG play a key role in the sensation of pain, inflammation in glial and neuronal cells and cell death in the DRG could also contribute to pain in LNB. Understanding the cross-talk between neuronal and glial cells of the PNS may prove useful in elucidating the mechanisms involved in Lyme peripheral neuropathy.

Unlike dexamethasone [32], meloxicam was unable to affect the levels of CCL2, IL-6, and IL-8 produced by rhesus DRG cell cultures in the presence of live Bb. These results support our in vivo findings of significantly reduced levels of inflammatory mediators, DRG-apoptosis, and lack of inflammatory neurodegenerative lesions in the nerve roots and DRG of Bb-infected animals that were treated with dexamethasone, but not in those that were treated with meloxicam [31].

An important finding of this study is that live Bb can induce several cytokines and chemokines in HSC (Table 1), and that dexamethasone but not meloxicam significantly reduces all inflammatory mediators, in addition to cell death in HSC. Interestingly, dexamethasone treatment resulted in increased level of the anti-inflammatory cytokine IL-10 as compared to that induced by Bb alone. This finding suggests a mechanism of action for this drug’s anti-inflammatory properties in our system. In addition, dexamethasone has been reported to down-regulate pro-inflammatory cytokines like INF-γ and TNF, while up-regulating the levels of IL-10 in peripheral blood mononuclear cells from uveitis patients [44].

The relative increase in the levels of certain immune mediators observed in HSC stimulated with live Bb in the presence of 100 μM meloxicam (Table 1) as compared to that induced by Bb alone may be due to decreased levels of apoptosis in the presence of meloxicam. We detected a protective effect of meloxicam on satellite-glial cell apoptosis in the DRG of rhesus macaques inoculated in vivo with live Bb after a prolonged time (14 weeks) post-inoculation [31].

The potential of Schwann cells to initiate the process of Wallerian degeneration by releasing pro-inflammatory cytokines involved in leukocyte recruitment including CCL2, IL-8, and IL-6 has been documented [45]. The cytokines and chemokines induced by Bb in Schwann cells that we report here could contribute to mediating inflammatory and apoptotic signaling cascades in these cells and trigger mechanisms of demyelination in the PNS similar to those of other demyelinating diseases [46, 47]. They could also contribute to the neuritis, axonal degeneration, and axonal regeneration seen in peripheral LNB [9, 48–52]. The importance of cytokine/chemokine signaling and apoptosis in the regulation of inflammatory responses in neurodegenerative diseases is well documented [53, 54].

The mechanism of action of meloxicam centers on the inhibition of COX-1 and, primarily, COX-2 [34, 55], both of which are key enzymes in the process of synthesis of prostaglandins (which are mediators of inflammation). The drug itself is a less potent anti-inflammatory as compared to dexamethasone and some other NSAIDS [56]. A specific role for COX-2 in Bb-induced inflammation and the potential of meloxicam to modulate this effect remains to be ascertained.

While treatment with antibiotics is the first line of defense, adjuvant treatment with steroids of patients and animals with Lyme borreliosis has been performed, with both beneficial [31, 57–60] and harmful outcomes [61, 62]. Therefore, the implications of the findings of the present study with regard to the treatment of human disease are not clear. What is known, however, is that dexamethasone modulates numerous signaling cascades that in turn regulate both inflammation as well as apoptosis [33, 63, 64]. Thus, it is possible that these signaling pathways play a role in mediating both inflammation and apoptosis as a result of *B. burgdorferi* infection in vivo*.* Evaluating the role of the signaling mechanisms that contribute to the anti-inflammatory potential of dexamethasone in the context of LNB could serve to identify potential therapeutic targets for limiting disease manifestations.

## Conclusions

Our results indicate that while steroids could be effective in curbing the inflammatory responses to *B. burgdorferi* in the PNS, this is not the case for NSAIDs such as meloxicam. They also pave the way towards elucidating the inflammatory mechanisms that are elicited by the Lyme disease spirochete in the PNS. Such mechanisms must be at least in part, those that are inhibited by dexamethasone, e.g., MAPK pathways, and likely not those that involve the action of COX.

## Abbreviations

Bb: *Borrelia burgdorferi*
BSK: Barbour-Stoenner-Kelly
COX: cyclooxygenase
CSF: cerebrospinal fluid
ELISA: enzyme-linked immunosorbent assay
LNB: Lyme neuroborreliosis
MBP: myelin basic protein
MOI: multiplicity of infection
MTT: 3-(4,5-dimethylthiazol-2-yl)-2,5-diphenyltetrazolium bromide
NSAID: non-steroidal anti-inflammatory drug
PBS: phosphate-buffered saline
PNS: peripheral nervous system
TUNEL: terminal deoxynucleotidyl transferase dUTP nick end labeling
